# Tissue-specific cytokine release from human extra-placental membranes stimulated by lipopolysaccharide in a two-compartment tissue culture system

**DOI:** 10.1186/1477-7827-7-117

**Published:** 2009-10-26

**Authors:** Natalie W Thiex, Mark C Chames, Rita K Loch-Caruso

**Affiliations:** 1 Department of Environmental Health Sciences, School of Public Health, The University of Michigan, 1415 Washington Heights, Ann Arbor, MI 48109-2029, USA; 2 Department of Obstetrics and Gynecology, University of Michigan Health System, F4835 Mott Hospital, Ann Arbor, MI 48109-0264, USA

## Abstract

**Background:**

The extra-placental gestational membranes secrete cytokines in response to bacteria and other infectious agents, with potentially adverse consequences for pregnancy. The present study used lipopolysaccharide (LPS) as a prototype endotoxin to investigate the pattern of stimulated cytokine release from the amniotic and choriodecidual sides of full-thickness human gestational membranes in a two-compartment tissue culture system.

**Methods:**

Gestational membranes were collected from healthy non-laboring caesarean deliveries at term. Full-thickness membranes from each placenta were cut into pieces, mounted on Transwell frames, and placed in culture wells to create a two-compartment culture with the gestational membranes serving as the barrier between compartments. The LPS (100 ng/ml) was added to the amniotic, choriodecidual or both chambers of the culture, and cytokines were assayed in the medium of the amniotic and choriodecidual chambers after 8 h of LPS exposure. Cytokine concentrations were analyzed by two-way analysis of variance for effects of treatment and side specificity of cytokine release from the membranes.

**Results:**

LPS exposure on the choriodecidual side of the membranes significantly increased TNF-alpha, IL-6, IL-10 and IL-8 in the choriodecidual compartment, whereas TNF-alpha was the only cytokine observed to increase in the amniotic compartment. When LPS treatment was to the amniotic side of the membranes, there were significant increases in TNF-alpha and IL-6 in the amniotic compartment as well as increased concentrations of TNF-alpha, IL-6 and IL-8 in the choriodecidual compartment; however, there were no statistically significant differences for IL-10 in either compartment. No statistically significant differences were observed for IL-1beta, TGF-beta or IL-4 concentrations in response to LPS, regardless of the exposure modality.

**Conclusion:**

The amnion and choriodecidua exhibited distinct patterns of response to LPS with evidence of inflammatory signaling across the layers of the gestational membranes. These results suggest a complicated network of signaling within the gestational membranes, in which cytokine- and tissue-specific responses to inflammatory stimulation may have important implications for maintaining pregnancy in the challenge of microbial invasion of the uterine compartment.

## Background

Although cytokines were initially described as immune cell messengers, cytokine expression occurs in non-immune cells, including cells and tissues from extra-placental gestational membranes [1]. In amniotic fluid, interleukin (IL)-1β, IL-6, IL-8 and tumor necrosis factor-α (TNF-α) concentrations increase after the onset of labor [2]. Similarly, IL-6 and TNF-α are detected in cervico-vaginal fluids in the third trimester of pregnancy before the onset of labor and increase after the onset of labor [3]. Intrauterine cytokines play important roles in parturition by stimulating cervical dilation and rupture of membranes through induction of prostaglandins [4-7] and matrix metalloproteinases [7-9].

Gestational tissues collected from preterm births show overt signs of microbial infection in 25-40% of cases [10]. Intra-uterine microbial infection stimulates an immune response with mobilization of immune cells and release of inflammatory cytokines. Elevated amniotic and cervico-vaginal fluid concentrations of the inflammatory cytokines IL-1β, IL-6, IL-8 and TNFα are associated with reproductive tract infection and preterm birth [11-16], as well as other adverse birth outcomes [17].

Lipopolysaccharide (LPS) is a cell membrane component from gram negative bacteria that elicits a strong immune response and is commonly used as a clinically relevant model inflammatory agent. Under conditions of intrauterine infection, LPS may be an important signaling molecule for preterm birth [18]. Injection of LPS into the peritoneum [19,20] or cervix [21] causes premature expulsion of fetuses in mouse models of preterm labor and birth. Similarly, intra-amniotic infusion of LPS induces preterm labor in sub-human primates [22]. The present study used LPS as a model inflammatory agent to investigate the patterns of TNF-α, IL-1β, IL-4, IL-6, IL-8, IL-10 and TGF-β release from amnion and choriodecidua of full-thickness human gestational membranes in Transwell cultures. We hypothesized that the amnion and choriodecidua respond in distinct manners to LPS with evidence of inflammatory signal transduction across the layers of the extra-placental gestational membranes.

## Methods

### Chemicals, reagents and antibodies

Tissue culture reagents including high glucose Dulbecco's Modified Eagle's Medium (DMEM) with no phenol red, penicillin/streptomycin antibiotic, and heat-inactivated fetal bovine serum were purchased from Invitrogen (Carlsbad, CA). We used lipopolysaccharide (LPS) from *Salmonella typhimurium *(Lot #225 purchased from List Biological Laboratory, Campbell, CA) as a model stimulus to ensure a strong inflammatory response. Transwell insert frames without synthetic membranes were a gift from Corning Corporation (Corning, NY).

### Tissue collection

Methods pertaining to human tissue were reviewed and approved by the University of Michigan Institutional Review Board (IRB) prior to initiation of experiments; review and approval were updated yearly. Full-thickness gestational membranes comprised of amnion, chorion and decidua were collected from non-laboring women with intact membranes at 37-39 weeks who underwent elective caesarean deliveries following healthy pregnancies. Membranes from seven women were used in the concentration-response experiment and membranes from five women were used to assess side specific responses. Exclusion criteria included smoking, multi-fetal gestation, complications of pregnancy such as gestational diabetes or hypertension, use of anti-inflammatory drugs, or any condition that would require the tissue to be sent to pathology. The inclusion and exclusion criteria were applied to minimize variability between sample responses; however, differences in gestational age or proximity to parturition still may have contributed to variation. Within 1-30 min after delivery, full-thickness extra-placental membranes were dissected from the placenta with scissors, placed in phosphate buffered saline solution, and transported to the laboratory.

### Tissue culture

Under sterile laboratory conditions, membranes were checked for integrity to ensure that the amnion and choriodecidua remained attached to one another. Culture medium was comprised of DMEM with 1% heat inactivated-fetal bovine serum (FBS), 100 units/ml penicillin, and 100 μg/ml streptomycin. Preliminary experiments were conducted with a range of FBS concentrations to determine optimal FBS concentrations in culture medium. In our system of using the membranes within 24 h of collection, we found that 1% FBS was sufficient to maintain the health of the tissue; no additional benefit was seen at higher FBS concentrations. The membranes were rinsed with culture medium to remove residual adherent blood. The membranes were cut into approximately 2 × 2 cm^2 ^pieces and affixed by elastic latex bands onto ethylene oxide-sterilized Transwell frames (without synthetic membrane) to create two distinct chambers on either side of the mounted membranes [23]. The choriodecidual side of the membranes faced the inner chamber of the Transwell device. Extra tissue was removed with a scalpel. Each Transwell frame with attached tissue was placed in a single well of a 12-well tissue culture plate with culture medium on both sides of the membranes. Membranes were equilibrated in culture medium in a 5% CO_2 _tissue culture incubator for 18-24 h, with a medium change after the first 2-4 h. The next day, the overnight culture medium was removed and the membranes were pretreated with fresh culture medium for 1-2 h.

### Experimental design

Culture wells with mounted gestational membranes were assigned randomly to one of the following treatment groups: untreated controls, LPS exposure on the amniotic side only, LPS exposure on the choriodecidual side only, or LPS exposure on both the amniotic and choriodecidual sides. Treatments were balanced across subjects, with membranes from each placenta divided among the treatments. To compensate for potential variability among cultures derived from different parts of the same placenta, three cultures per treatment were established from each placenta. Cultures from each subject comprised an experiment run on a separate day, with triplicate samples per treatment. Results from the treatment triplicates were averaged and used as the measure for that subject, with *n *referring to the number of subjects in each experiment.

### Treatment

Lipopolysaccharide was diluted in sterile, deionized water to make a primary stock solution of 100 μg LPS/ml. Just prior to addition to the cultures, the LPS primary stock solution was further diluted with culture medium (DMEM plus 1% FBS and antibiotics) to achieve the necessary experimental concentrations of LPS. The medium was then exchanged, with the LPS exposure medium added to one or both chambers of the Transwell culture. A concentration-response experiment was conducted with LPS concentrations of 1, 10, 100 and 1000 ng/ml in both the amniotic and choriodecidual compartments for TNF-α and IL-6. Based on the concentration-response experiment, the LPS concentration of 100 ng/ml was used to assess side-specific responses to LPS. For the latter side-specific response experiment, cultures treated with LPS on only one side of the membranes had fresh culture medium without LPS exchanged in the opposite chamber. Cultures were exposed to LPS for 8 h, an exposure duration that produced a robust response to TNF-α and IL-6 in preliminary experiments. After the 8-h treatment, medium was recovered from each side of the membranes and stored at -80°C for subsequent enzyme immunoassay analysis of cytokines. After collection of medium, the tissue was removed from the Transwell frame and weighed.

### Cytokine enzyme-linked immunosorbant assays

Cytokine sandwich enzyme-linked immunosorbant assays (ELISAs) for TNF-α, IL-1β, IL-4, IL-6, IL-8, IL-10, and TGF-β were conducted by the University of Michigan Cellular Immunology Core Facility. DuoSet ELISA Development Systems assay kits specific for each cytokine were performed in duplicate according to the manufacturer's protocol (R & D Systems, Minneapolis, MN). The assay buffer used was 0.2% casein in tris-buffered saline. Cytokine concentrations (pg/ml) were divided by the wet weight of the gestational membrane tissue to adjust for any differences in tissue thickness between samples. The mean cytokine concentration per gram tissue of the treatment triplicates was calculated and used as the measure for that subject.

### Statistical analyses

The cytokine enzyme immunoassay results were analyzed by two-way repeated measures analysis of variance (ANOVA) to test for effects of LPS treatment, gestational membrane side specificity and interaction between these main effects using SigmaStat v 3.5 software (Systat Software, Inc., Richmond, CA). The data were matched by Transwell culture for cytokine measures from the amniotic and choriodecidual compartments for the ANOVA. For TNF-α and IL-6, the data were transformed prior to ANOVA using log normal transformation to improve normality and stabilize the variances. Post hoc pair-wise comparisons of means were performed by the Student-Newman-Keuls test. A *p*-value ≤ 0.05 was considered statistically significant.

## Results

### Concentration-response of LPS-stimulated TNF-α and IL-8 release

**Figure 1:**
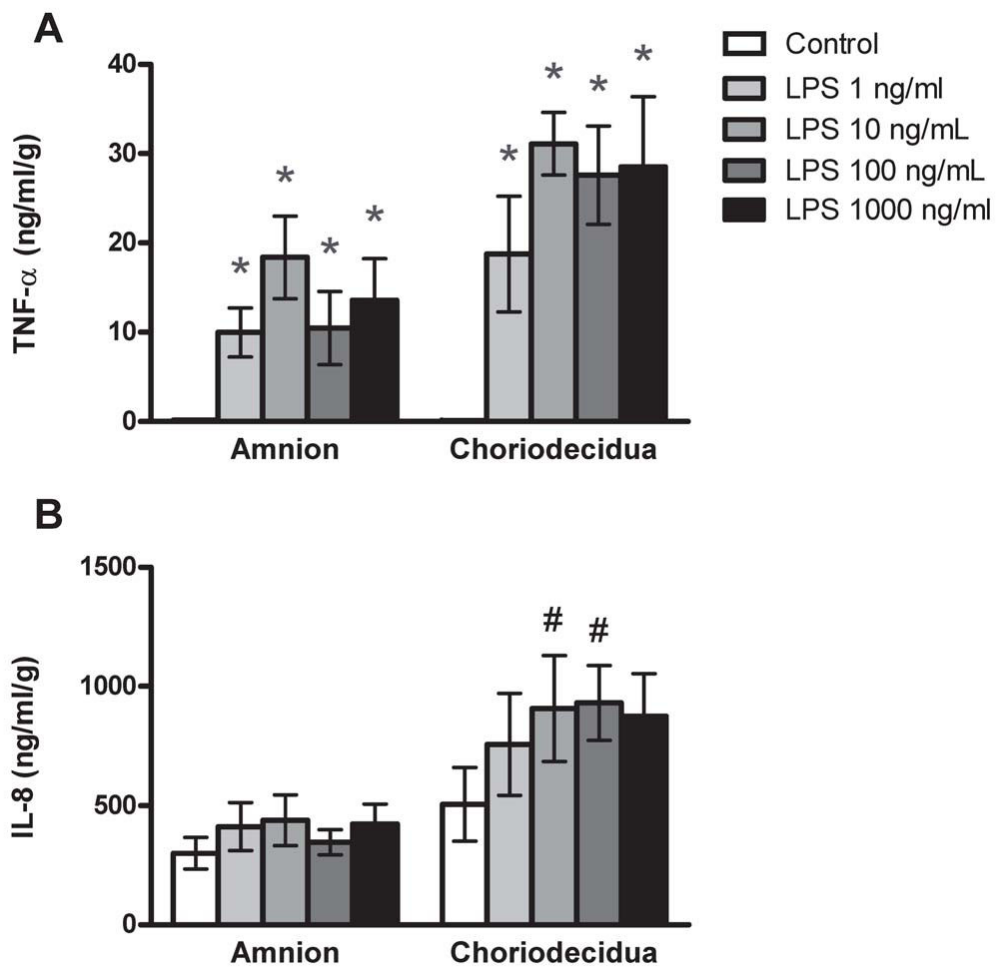
**Concentration-response of lipopolysaccharide-stimulated release of TNF-α and IL-8 from human gestational membranes in Transwell cultures**. Cytokine concentrations were measured in medium from the amniotic and choriodecidual sides of Transwell cultures of human extra-placental gestational membranes after an 8-h exposure to 1, 10, 100 or 1000 ng LPS/ml. The LPS was added to both the amniotic and choriodecidual compartments of the cultures. Controls were exposed to culture medium without LPS. The very low means of TNF-α concentrations in controls are nearly contiguous with the x-axis. Values are expressed as mean ± SEM of combined values from triplicate cultures for each subject; *n *= 7 subjects. *Significantly different from control values from the same side of the membranes (*p *< 0.05). ^#^Significantly different from amniotic compartment values of the same treatment (*p *< 0.05).

Treatment with LPS significantly elevated TNF-α concentrations in cultures exposed for 8 h to 1, 10, 100 or 1000 ng LPS/ml on both the amniotic and choriodecidual sides of the membranes (Fig. 1A; ANOVA LPS treatment effect, *p *< 0.001). TNF-α concentrations in the amniotic and choriodecidual compartments were significantly elevated at all concentrations of LPS compared with controls (P < 0.05). In the same cultures, concentrations of IL-8 were elevated in the choriodecidual compartment compared with the amniotic compartment of cultures (Fig. 1B; ANOVA membrane side effect, *p *< 0.001), with significantly increased IL-8 concentrations in the choriodecidual compartment compared with the amniotic compartment at 10 and 100 ng LPS/ml (p < 0.05). However, IL-8 differences between LPS-treated groups and controls were not statistically significantly different. Moreover, there were no statistically significant differences between LPS concentrations in pairwise comparisons for either cytokine and no trend toward an increased response was observed between 10 and 1000 ng LPS/ml, indicating an apparent plateau in the TNF-α and IL-6 responses. Based on these concentration-response results, we chose 100 ng/ml as the LPS concentration for further experiments because this concentration produced a maximal response. A previous report used the higher concentration of 500 ng LPS/ml in the Transwell culture system with human gestational membranes [23].

### Side-specific LPS-stimulated TNF-α release

**Figure 2:**
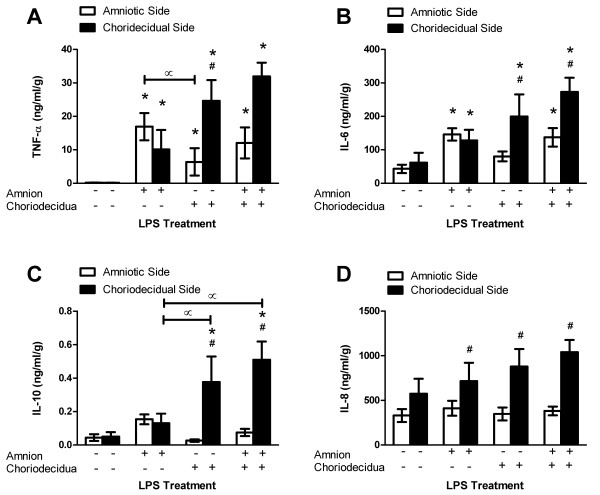
**Lipopolysaccharide-stimulated release of cytokines from human gestational membranes in Transwell cultures**. Cytokine concentrations were measured in medium from the amniotic and choriodecidual sides of Transwell cultures of human extra-placental gestational membranes after an 8-h exposure to 100 ng/ml LPS on the amniotic side only, choriodecidual side only, or both sides of the membranes. Controls were exposed to culture medium without LPS. For some cytokines the very low means of controls are nearly contiguous with the x-axis. Values are expressed as mean ± SEM of combined values from triplicate cultures for each subject; *n *= 5 subjects. *Significantly different from control values of medium from the same side of the membranes (*p *< 0.05). ^#^Significantly different from amniotic compartment values of the same treatment (*p *< 0.05). ^∝ ^Significantly different between LPS treatments (*p *< 0.05).

Nominal TNF-α was detected in the medium of control gestational membrane cultures, yet LPS strongly stimulated TNF-α release from both the amniotic and choriodecidual sides of the membranes regardless of whether LPS treatment was on the amniotic side only, choriodecidual side only or both sides simultaneously (Fig. 2A; ANOVA LPS treatment effect, *p *< 0.001; pairwise comparisons with membrane side control, p < 0.05). Notably, TNF-α was released from the side of the membranes opposite to the side exposed to LPS (without direct LPS exposure) as well as on the side exposed directly to LPS. The pattern of TNF-α response was different for the amniotic versus the choriodecidual side of the membranes depending on the treatment modality (ANOVA treatment * membrane side interaction, *p *= 0.004). Specifically, TNF-α concentrations were significantly higher in the amniotic compartment medium compared with the choriodecidual compartment medium when LPS was added to the amniotic side of the Transwell cultures, whereas TNF-α concentrations were significantly higher on the choriodecidual side compared to the amniotic side when LPS exposure was to the choriodecidual side of the membranes (*p *< 0.05). Likewise, TNF-α concentrations in the amniotic compartment were higher when LPS was added to the amniotic side only versus the choriodecidual side only (p < 0.05). Cultures exposed to LPS via both the amniotic and choriodecidual compartments exhibited elevated concentrations of TNF-α on the choriodecidual side that approached but did not achieve statistical significance compared with the amniotic side (*p *= 0.052).

### Side-specific LPS-stimulated IL-6 release

Exposure to LPS stimulated increased IL-6 release from the amniotic and choriodecidual sides of the membranes (Fig. 2B; ANOVA LPS treatment effect, *p *= 0.008), but the pattern of IL-6 response was different for the amniotic versus the choriodecidual side of the membranes depending on the treatment modality (ANOVA treatment * membrane side interaction, *p *= 0.03). LPS treatment of only the amniotic side of the membranes increased IL-6 concentrations to a similar extent in the medium of both the amniotic and choriodecidual compartments compared with respective untreated controls (*p *< 0.05). In contrast, LPS treatment of only the choriodecidual side of the membranes significantly increased IL-6 concentrations in the medium of only the choriodecidual compartment compared with respective untreated control and compared with the amniotic side response (*p *< 0.05). When LPS treatment was to both the amniotic and choriodecidual sides of the membranes, the IL-6 concentrations increased in the medium of both the amniotic and choriodecidual compartments with respect to untreated controls (*p *< 0.05), but to a greater extent on the choriodecidual side compared with the amniotic side (*p *< 0.05). These results show that LPS treatment of only the amniotic side of the gestational membranes stimulated a similar IL-6 response on the amniotic and choriodecidual sides of the membranes, but that the converse was not true for LPS treatment of only the choriodecidual side which stimulated increased IL-6 release on the choriodecidual side only.

### Side-specific LPS-stimulated IL-10 release

Treatment with LPS had no statistically significant effect on IL-10 release from the amniotic side of the membranes but stimulated release from the choriodecidual side (Fig. 2C; ANOVA side specificity effect, *p *< 0.001; ANOVA treatment effect, *p *= 0.036; ANOVA treatment * membrane side interaction, *p *= 0.005). Specifically, LPS exposure on the choriodecidual side, either alone or in combination with exposure on the amniotic side, produced significant increases in IL-10 concentrations on the choriodecidual side compared with controls and compared with IL-10 concentrations released into the amniotic compartment (*p *< 0.05). In addition, concentrations of IL-10 were higher in the choriodecidual compartment when LPS was added to the choriodecidual compartment compared with cultures exposed to LPS via the amniotic compartment only (*p *< 0.05). When both sides of the membranes were treated with LPS, the choriodecidual response was approximately 10-fold higher than control levels. These data indicate that the choriodecidua is the dominant producer of IL-10 in human gestational membranes in this system.

### Side-specific LPS-stimulated IL-8 release

Unstimulated control cultures released substantial amounts of IL-8 in the Transwell cultures on both the amniotic and choriodecidual sides of the gestational membranes (Fig. 2D). Moreover, significantly greater concentrations of IL-8 were detected in the medium on the choriodecidual side of the membranes compared with the amniotic side in cultures treated with LPS (ANOVA membrane side specific effect, *p *< 0.001; pairwise comparisons, *p *< 0.05). However, mean differences between the LPS-stimulated IL-8 concentrations in the choriodecidual compartment medium were not statistically significantly different from the mean choriodecidual medium concentration of unexposed controls. These results suggest that the choriodecidua is the dominant contributor of IL-8 in this culture system, particularly under conditions of LPS stimulation.

### Side-specific LPS-stimulated IL-1β, TGF-β and IL-4 release

No statistically significant differences were observed for IL-1β, TGF-β or IL-4 release from the amnion or choriodecidual side of gestational membranes in response to treatment with 100 ng/ml LPS for 8 h (data not shown).

## Discussion

**Figure 3:**
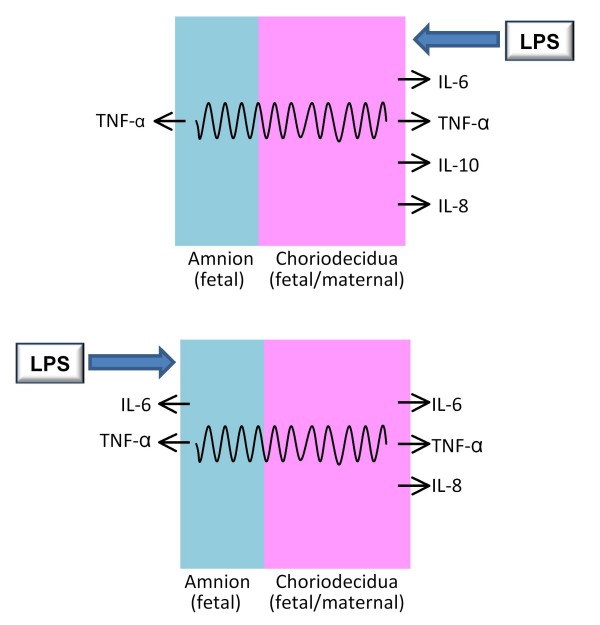
**Summary of LPS-stimulated cytokine release from amnion and choriodecidua in Transwell cultures**. Cytokines were released in a distinct manner from the amniotic and choriodecidual sides of human extra-placental gestational membranes exposed to LPS in Transwell cultures. **Top**: Exposure to LPS on the choriodecidual side of the membranes stimulated choriodecidual release of IL-6, TNF-α, IL-10 and IL-8; TNF-α release increased on the amniotic side, also. **Bottom**: Exposure to LPS on the amniotic side of the membranes stimulated amniotic release of IL-6 and TNF-α; IL-6, TNF-α, IL-10 and IL-8 increased on the choriodecidual side, also. The wavy lines indicate inflammatory signal transduction across the membranes by an unknown mechanism.

The extra-placental gestational membranes are an important source of cytokines during pregnancy [1]. Using a two-compartment culture system in which full-thickness human gestational membranes were mounted on Transwell frames, we observed distinct patterns of cytokine release from the amniotic and choriodecidual sides of the membranes. Tumor necrosis factor-α and IL-6 concentrations increased significantly on the amniotic and choriodecidual sides of the membranes after an 8-h exposure of the membranes to LPS (100 ng/ml). In contrast, IL-8 and IL-10 increased only on the choriodecidual side of the membranes. This side-specific pattern of cytokine response is illustrated in Figure 3. Our findings confirm a previous report on LPS-stimulated release of TNF-α from human gestational membranes in Transwell cultures [23], and show for the first time that releases of IL-6, IL-8 and IL-10 are stimulated by LPS in a side-specific manner from human gestational membranes.

The side-specific cytokine responses seen with ex vivo tissue exposure to LPS are consistent with cytokine production observed in women during parturition. Increased TNF-α and IL-6 concentrations in medium from the amniotic side of the membrane culture are consistent with detection of TNF-α and IL-6 in amniotic fluid from women in preterm or term labor [1]. Likewise, IL-6 and IL-8 are detected in cervico-vaginal fluids in cases of spontaneous term parturition and preterm labor [1], and we measured these cytokines in medium from the choriodecidual side of the membranes. Amniotic fluid IL-6, in particular, has emerged as a sensitive indicator of intra-amniotic infection [24,25]. Our findings suggest that an elevated concentration of IL-6 in the amniotic fluid may be a specific marker of infection/inflammation in the amniotic cavity, whereas infection/inflammation isolated to the maternal compartment may not be sufficient to elevate amniotic fluid IL-6 concentrations. Additional clinical/epidemiologic experiments beyond the scope of this study will be required to confirm the latter suggestion.

This ability of LPS exposure on one side of the membranes to stimulate release of cytokines from the opposite side provides evidence of inflammatory signal communication across the gestational membranes, as indicated in Figure 3. Our results with IL-6 and IL-8, that exposure to LPS on the amnion side stimulated IL-6 and IL-8 release on the choriodecidual side, provide new examples of inflammatory cytokine signaling across the gestational membranes. Our findings with TNF-α agree with those of Zaga et al., who showed increased LPS-simulated TNF-α release by both the amniotic and choriodecidual membranes regardless of whether LPS exposure was to the amniotic or choriodecidual side of human gestational membranes in a Transwell culture [23]. The patterns of cytokine release from human gestational membranes exposed to Group B streptococcus, *Candida albicans *or *Escherichia coli *in Transwell cultures support inflammatory signaling between the amnion and choriodecidua, although the pattern varies with microorganism [23,26,27].

Because TNF-α, IL-6 and IL-8 increased on the opposite side of the membranes when only one side was exposed to LPS, the TNF-α, IL-6 and IL-8 could have been released on one side of the membranes and moved through the membranes to the opposite side. In support of this explanation, both IL-6 [28] and TNF-α [29] cross the blood-brain barrier in mice. However, the rate of transfer of radiolabeled IL-6 and TNF-α across human extraplacental membranes is low, less than 17% and 7%, respectively, after 24 h in vitro [30], suggesting that transfer of the cytokine cannot solely explain the contralateral responses observed for IL-6 and TNF-α. Moreover, LPS treatment of the amnion side failed to increase IL-10 or IL-8 on the amniotic side of the membranes yet increased IL-10 and IL-8 on the choriodecidual side, indicating that the inflammatory signal, but not the cytokine itself, most likely propagated across the membranes from the amnion to the choriodecidua. The mechanism by which an inflammatory signal would traverse the gestational membranes is not known. Considering the complexity of the gestational membrane tissue, a multiple-step transduction mechanism is suggested in the propagation of the inflammatory signal from one side of the gestational membranes to the other.

Among the cytokines measured in the present study, TNF-α exhibited the greatest relative response to LPS with mean concentrations 60-300 times higher than unstimulated controls. Similar LPS-stimulated increases were observed in a previous study, with the relative TNF-α increases exceeding IL-1β increases for membranes in Transwell cultures treated with LPS in a side-specific manner [23]. In another study with the Transwell system, TNF-α increased modestly on the amniotic side in response to addition of *E. coli *to the amniotic chamber, but a much stronger response was observed on the choriodecidual side when both sides of the membranes were treated with bacteria [26].

Lipopolysaccharide-stimulated IL-8 concentrations were at most two-fold higher than control concentrations, and were increased on the choriodecidual side of the membranes only. The absence of LPS-stimulated IL-8 release on the amniotic side of the membranes is in contrast to an earlier report showing that LPS increases IL-8 secretion from isolated amnion cells as well as from decidual cells [31]: these differences in response could reflect differences between cell and intact tissue cultures. In addition, Transwell cultures of human gestational membranes treated with *E. coli *increased release of IL-8 on both sides of the membranes [26]. The differences between the present study and latter study may be related to the use of LPS versus *E. coli *as an inflammatory stimulus, because different patterns of cytokine release from human gestational membranes were observed with exposure to Group B streptococcus, *C. albicans *or *E. coli *in Transwell cultures [23,26,27].

Lipopolysaccharide-stimulated IL-6 concentrations were approximately 4-fold higher than non-stimulated levels. Similar IL-6 secretion patterns are seen in Transwell cultures of human gestational membranes in response to side-specific *E. coli *exposure [26]. Because of the high variability of IL-1β release in response to LPS, we lacked sufficient power to detect statistically significant differences with our sample size. In contrast, Zaga et al. [23] saw a robust IL-1β response on both the amniotic and choriodecidual sides of the membranes exposed to LPS in Transwell cultures. Differences between our results and those of Zaga et al. [23] may be related to the higher LPS concentration and longer duration of exposure used by Zaga et al. (500 ng LPS/ml for 24 h). Similarly, the lack of LPS-stimulated IL-4 and TGF-β responses in our system should be interpreted with caution, and could be due to the specific culture conditions of our experiment. Although higher concentrations of LPS (1000 ng/ml) failed to stimulate significant release of IL-4 and TGF-β after 8 h of exposure in limited experiments (*n *= 2-3 subjects), longer durations of exposure may have yielded different results.

Culture of full-thickness gestational membranes may provide a more realistic approximation of an in vivo response than a single cell culture because inflammatory responses can be integrated among multiple tissues and cell types. In addition, the use of a Transwell insert, which maintains separate amniotic and choriodecidual compartments, is more physiologic than floating explant cultures that allow amniotic and choriodecidual sides of the membranes to share the same compartment. However, the use of full thickness membranes had the disadvantage that inflammatory responses can vary due to differences in thickness, decidual density, and handling, even within the membranes from a single placenta. In preliminary experiments, we found that we reduced intrasubject variation by averaging results from three tissue samples/cultures to derive a single value for each treatment from each subject. Consequently, we applied the latter approach in the present study, using the averaged values in our statistical calculations, with sample size determined by the numbers of subjects (not individual cultures/wells).

Because membranes may respond differently to inflammatory stimuli at different stages of gestation, the use of gestational membranes from healthy pregnancies at term (after 37 weeks gestation) in the present study is a potential limitation for the application of these results to preterm birth. We chose to work with membranes from healthy term pregnancies because preterm cesarean deliveries are medically indicated and, consequently, the membranes are more likely to be compromised by pathological conditions. It is important to note that pathological conditions associated with preterm birth may alter responses to inflammatory stimuli such as LPS, and that the relevance of the responses observed in vitro in the present report for preterm birth will require further investigation.

Lipopolysaccharide is a highly immunogenic molecule found in the outer membrane of gram negative bacteria. The LPS immunogenicity differs among microorganisms due to differences in the sugar moieties. *Salmonella typhimurium *is a gram negative bacterium that causes gastroenteritis usually associated with ingestion of contaminated food. Although *Salmonella *has not been identified in cases of intrauterine infection associated with adverse birth outcomes, we chose to work with LPS from *Salmonella typhimurium *as a model stimulus because *Salmonella *is highly inflammatory and would stimulate a strong response for assessment of side-specific cytokine responses.

Cytokines play critical roles in parturition, including the regulation of prostaglandin availability through induction of enzymes necessary for prostaglandin synthesis (phospholipase A_2 _and prostaglandin-endoperoxide synthase 2 (also known as COX-2) and through decreased expression of the prostaglandin catabolism enzyme hydroxyprostaglandin dehydrogenase 15-(nicotinamide adenine dinucleotide) in gestational membranes [4]. Prostaglandins E_2 _and F_2α _stimulate uterine contraction and initiate cervical ripening and membrane rupture to promote parturition [32]. In addition to effects on prostaglandin synthesis, proinflammatory cytokines increase expression of matrix metalloproteinases that degrade the extracellular matrix of the cervix and gestational membranes to promote cervical dilation and rupture of the membranes [33].

Inflammatory cytokine responses are elevated in preterm birth associated with infection [2]. Most often, intrauterine microorganisms originate as ascending infections in which microorganisms present in the vagina traverse the cervix and colonize the decidua [34]. Microorganisms can cross the gestational membranes, in some cases resulting in intraamniotic and/or fetal infection [2]. In the Transwell cultures, direct exposure to LPS stimulated a greater response on the choriodecidual side than the amniotic side for release of TNF-α, IL-6, IL-8 and IL-10. A robust choriodecidual cytokine response to an ascending infection could suppress the microbial invasion, thereby preventing infection of the amnion and fetus.

Although IL-4, IL-10 and TGF-β are anti-inflammatory cytokines that are important for controlling inflammation, only IL-10 increased on the choriodecidual side in response to LPS in our culture system. The anti-inflammatory properties of IL-10 could serve to limit inflammatory cytokine responses in the choriodecidua in the presence of an ascending microbial infection from the vagina, thereby reducing the risk for activation of parturition. IL-10 null mutant mice are more susceptible to LPS-induced fetal loss (a model for preterm delivery) than wild-type mice, and administration of exogenous IL-10 protects both IL-10 knockout mice and wild-type mice from fetal loss [35]. The latter mouse studies suggest that up-regulation of IL-10 and its anti-inflammatory activities may be important for preventing preterm labor due to inflammation.

## Conclusion

The present study shows for the first time that LPS stimulates release of IL-8 and IL-10 from the choriodecidual side only in human gestational membranes in Transwell tissue cultures. Moreover, new examples of inflammatory cytokine signaling across the gestational membranes were identified, with IL-6 and IL-8 released from the opposite side of the membranes exposed to LPS. The latter results with IL-6 and IL-8 extend results from a previous report of inflammatory cytokine signaling across human gestational membranes for TNF-α release [23], which were confirmed by the present study. Among the acute phase inflammatory cytokines, TNF-α exhibited the greatest relative response from the choriodecidual side of the membranes. Release of IL-10 increased from the choriodecidua in response to LPS, but no significant response was observed for the other anti-inflammatory cytokines, IL-4 and TGF-β. Together, these results demonstrate a complicated network of intra-gestational membrane signaling in response to an inflammatory stimulus. Although the cytokine- and tissue-specific responses of the gestational membranes to inflammatory stimulation suggest important implications for maintaining pregnancy in the challenge of microbial invasion of the uterine compartment, additional experiments are required to determine whether these responses in term membranes in vitro are relevant to preterm birth.

## Competing interests

The authors declare that they have no competing interests.

## Authors' contributions

NWT conducted the Transwell culture experiments, analyzed the data and drafted the initial manuscript. RLC participated in the design of the study, analysis and interpretation of the statistical analysis, graphing of the data, and drafting of the final manuscript. MC participated in the design of the study, facilitated the acquisition of human tissues, and reviewed manuscript drafts. All authors read and approved the final manuscript.
